# Supplementary material for: Joint Effects of Exercise and Ramadan Fasting on Telomere Length: Implications for Cellular Aging
Source: Biomedicines. 2024 May 27;12(6):1182. doi: 10.3390/biomedicines12061182 (PMC11200901; doi:10.3390/biomedicines12061182)
Supplement: Supplementary file 1 [file biomedicines-12-01182-s001.zip › biomedicines-3016250-supplementary.pdf]

Supplementary Table S1. Characteristics of participants before and after training for groups 4W and 4W+F.

	4W			4W+F		
	Before	After	p-value	Before	After	p-value
Age	21 (20-26)			22 (20.75-22.25)		
Height (m)	1.6 (0.06)			1.59 (0.05)		
BMI	24.78 (5.87)	24.63 (5.76)	0.192	24.42 (4.54)	24.28 (4.5)	0.305
T/S ratio	0.335 (0.094)	0.328 (0.137)	0.870	0.284 (0.24-0.34)	0.393 (0.22-0.55)	0.048
Weight (Kg)	65.1 (49.1-71.3)	65.6 (47.9-70.6)	0.308	58.75 (51.8-65.72)	58.95 (51.53-64.9)	0.805
Body fat (%)	0.31 (0.11)	0.29 (0.11)	0.244	0.32 (0.09)	0.32 (0.09)	0.933
Fat free mass (Kg)	42.95 (4.65)	42.95 ± 3.94	1	43.85 (4.49)	43.77 (4.27)	0.709
Fat mass (Kg)	20.54 (11.68)	20.34 ± 12.00	0.585	21.86 (11.36)	24.17 (13.98)	0.215
muscle mass (Kg)	40.77 (4.43)	40.75 ± 3.75	0.971	41.59 (4.27)	41.55 (4.06)	0.832
MET	975 (622-1480)	1233 (958-1986)	0.305	1251.25 (826.5-2708.25)	1577.25 (1211-2094)	0.677
6WT Distance (m)	531 (62.71)	636.6 (79.71)	0.0008	587.83 (70.68)	716 (175.06)	0.057
Handgrip L	21.83 (4.04)	23.02 (5.73)	0.320	21.92 (5.6)	23.89 (5.68)	0.023
Handgrip R	23.44 (5.1)	25.38 (8.36)	0.329	22.18 (5.37)	24.77 (5.41)	0.021
Insulin (mU/L)	13.03 (7.69)	11.73 (4.92)	0.392	12.13 (5.13)	13.3 (4.66)	0.328
FBS (mmol/L)	5.1 (4.7-5.3)	5.0 (4.7-5.6)	0.368	5.3 (4.8-5.32)	5.4 (5.2-5.55)	0.061
HOMA-IR	2.39 (1.76-3.94)	2.89 (1.73-3.53)	1	3.73 (1.99-4)	2.37 (2.18-3.74)	0.969
Total Cholesterol (g/dl)	184 (159-202)	181 (167-198)	0.850	161.5 (147.75-174.5)	167.5 (152-183.5)	0.638
Triglycerides (g/dl)	64 (50-75)	57 (47-78)	0.484	59.5 (48.5-66)	53.5 (38-67)	0.266
HDL (g/dl)	68.31 (13.81)	65.85 ± 14.05	0.169	55.45 (14.7)	53.17 (14.6)	0.732
LDL (g/dl)	101.46 (24.37)	101.69 ± 24.61	0.953	101.92 (35.19)	109.42 (39.35)	0.182
HbA1C %	4.9 (4.7-5.5)	5.1 (4.8-5.4)	0.820	5.25 (4.82-5.38)	5.19 (5.05-5.31)	0.548
Total cholesterol: HDL ratio	2.76 (0.64)	2.85 (0.64)	0.210	3.09 (0.89)	3.47 (1.23)	0.756
SOD (U/ml)	0.97 (0.87-1.22)	1.13 (0.96-1.22)	0.184	0.92 (0.83-1.08)	0.99 (0.78-1.1)	0.784
Catalase (U/ml)	19.61 (18.78-19.92)	20.04 (19.82-20.24)	0.089	20.09 (18.69-20.21)	19.99 (19.33-20.29)	0.410
IL 1 beta (pg/ml)	0.14 (0.14-0.14)	0.14 (0.14-0.5)	0.499	0.14 (0.14-0.32)	0.14 (0.14-0.32)	0.999
IL 8 CXCL8 (pg/ml)	1.37 (0.83)	2.32 (2.59)	0.905	2.4 (1.72)	4.07 (2.9)	0.152
IL 1RA (pg/ml)	294.02 (176.92-607.44)	365.68 (237.64-525.73)	0.850	457.1 (204.11-1068.14)	521.25 (220.23-1098.94)	0.894
TNF alpha (pg/ml)	0.43 (0.43-3.95)	3.95 (3.95-3.95)	0.143	0.43 (0.43-3.95)	0.43 (0.43-3.95)	0.269
